# Supplementary material for: Sequence analysis of tyrosine recombinases allows annotation of mobile genetic elements in prokaryotic genomes
Source: Mol Syst Biol. 2021 May 20;17(5):e9880. doi: 10.15252/msb.20209880 (PMC8138268; doi:10.15252/msb.20209880)
Supplement: Supplementary file 1 — Appendix [file MSB-17-e9880-s007.pdf]

Table of Contents

Appendix Figure S1. Maximum likelihood phylogenetic tree of tyrosine recombinases (YRs). *2*

Appendix Figure S2. Sequence conservation of the N-terminal part of YR catalytic (CAT) domains. *3*

Appendix Figure S3. Sequence conservation of the central part of YR CAT domains. *4*

Appendix Figure S4. Sequence conservation of the C-terminal part of YR CAT domain *5*

Appendix Figure S5. Full sequence logos of the YRs from RitA, RitC and TnpA subgroups. *6*

Appendix Figure S6. Maximum-likelihood tree of integrases of ICEs and phages from the IntTn916 subgroup. *7*

Appendix Figure S7. Maximum-likelihood tree of integrases of ICEs and phages from the IntSXT subgroup. *8*

Appendix Figure S8. Structural composition of the ICEs. *9*

Appendix Figure S9. Verification of the ICE identification. *10*

Appendix Table S1. Statistical support for identified phylogenetic clades. *11*

Appendix Table S2. Benchmarking of e-values for jackhammer search. *12*

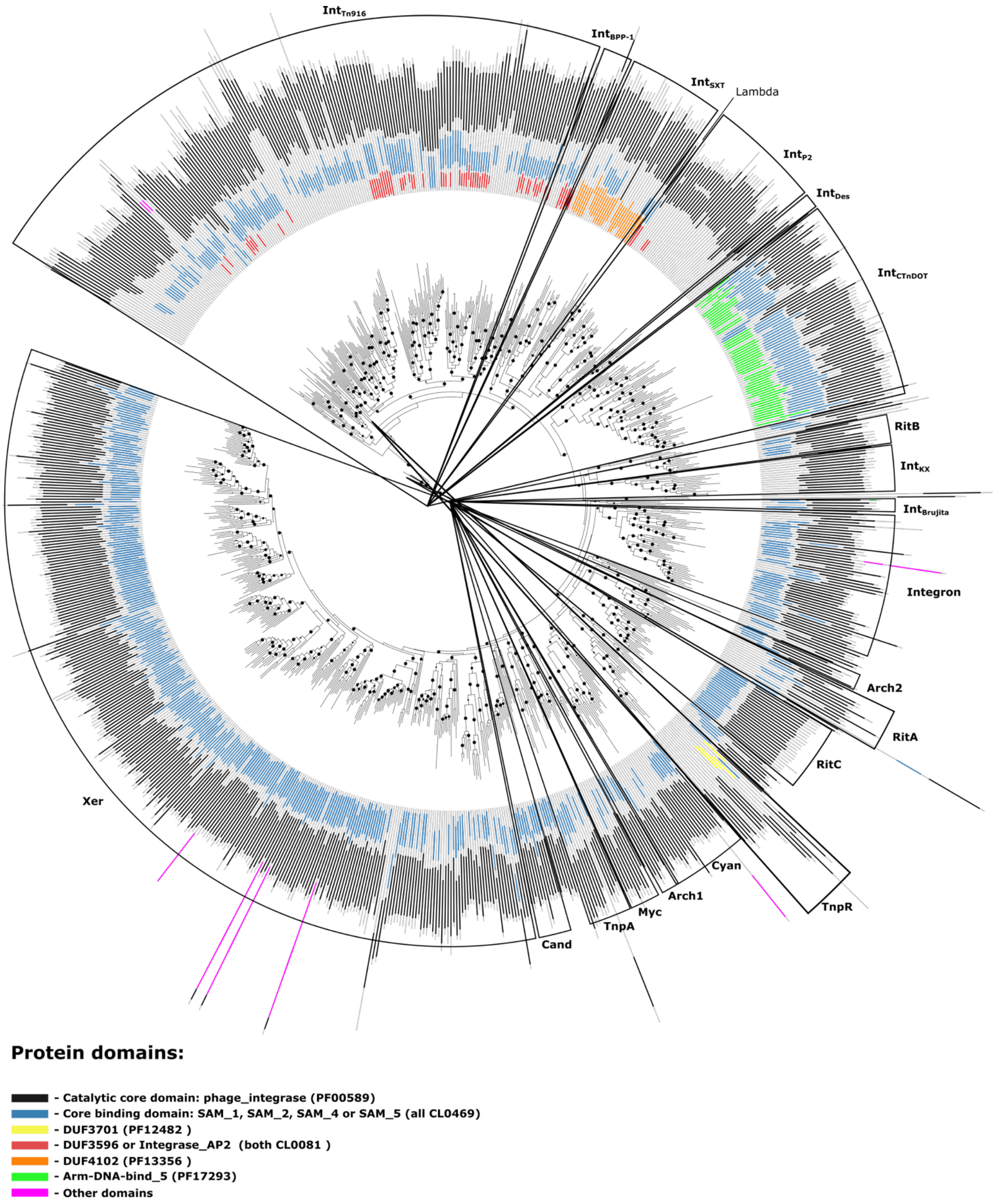

Appendix Figure S1. Maximum likelihood phylogenetic tree of tyrosine recombinases (YRs). Statistical support was evaluated by aBayes and values higher than 0.98 are shown as black circles at the corresponding nodes (the larger the circle the higher the value). Subgroups of the YRs are marked outside the tree. The domain architectures of the individual proteins are shown with colored bars representing the predicted domains (color code is shown at the bottom of the figure).

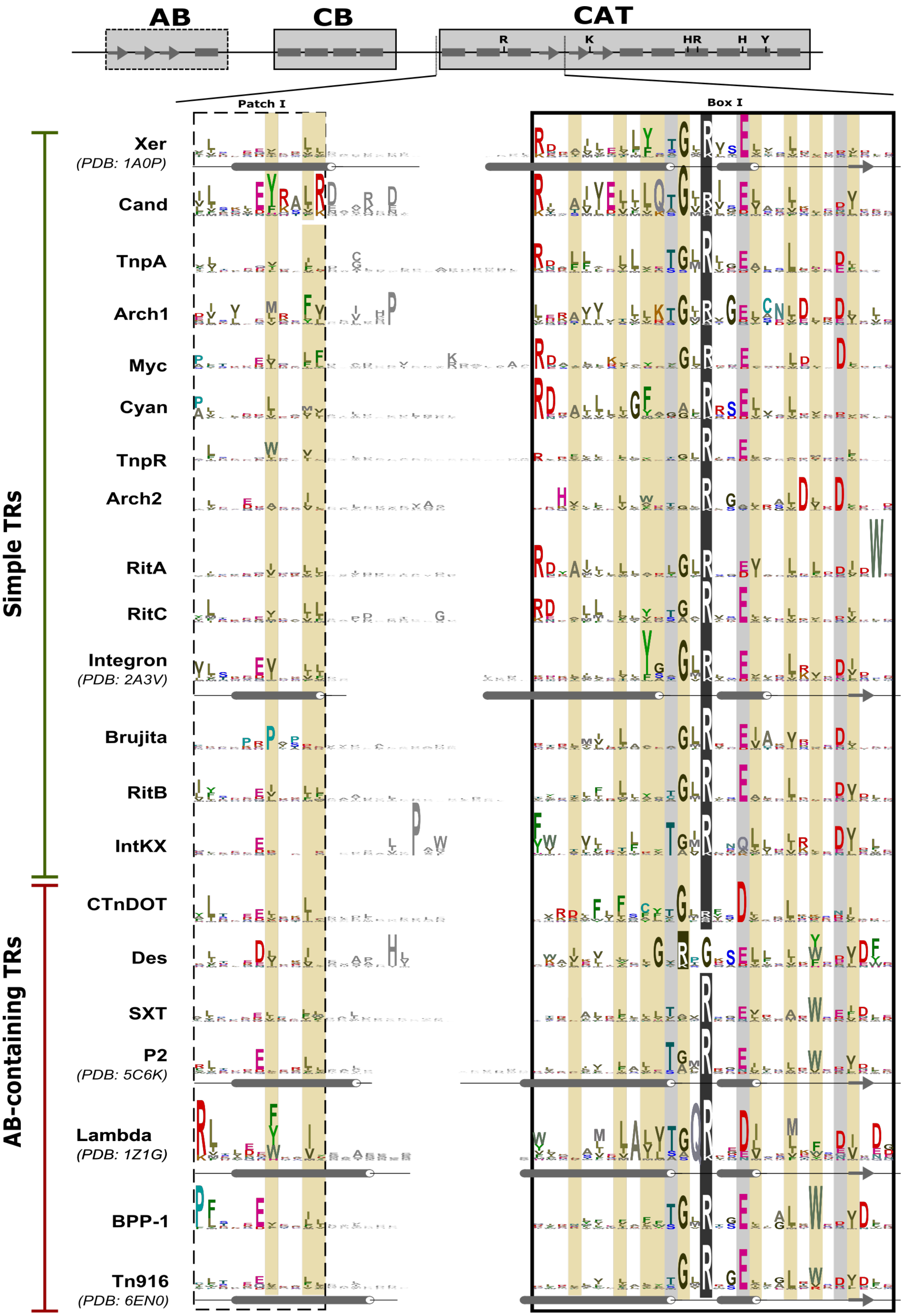

Appendix Figure S2. Sequence conservation of the N-terminal part of YR catalytic (CAT) domains. Manually aligned web logos were produced after HMM search against the UniProt reference proteomes database for each of the subgroups. Conserved boxes and patches were manually aligned and are colored by residue type. The tyrosine nucleophile and the catalytic RKHRH pentad are highlighted with black background. Positions corresponding to conserved residues located near the active site pocket are highlighted in grey. The conserved hydrophobic residues that form the core of the catalytic domain are highlighted in beige. The secondary structures of a representative subgroup member were retrieved from PDB entries, where available, and are shown below the logos.

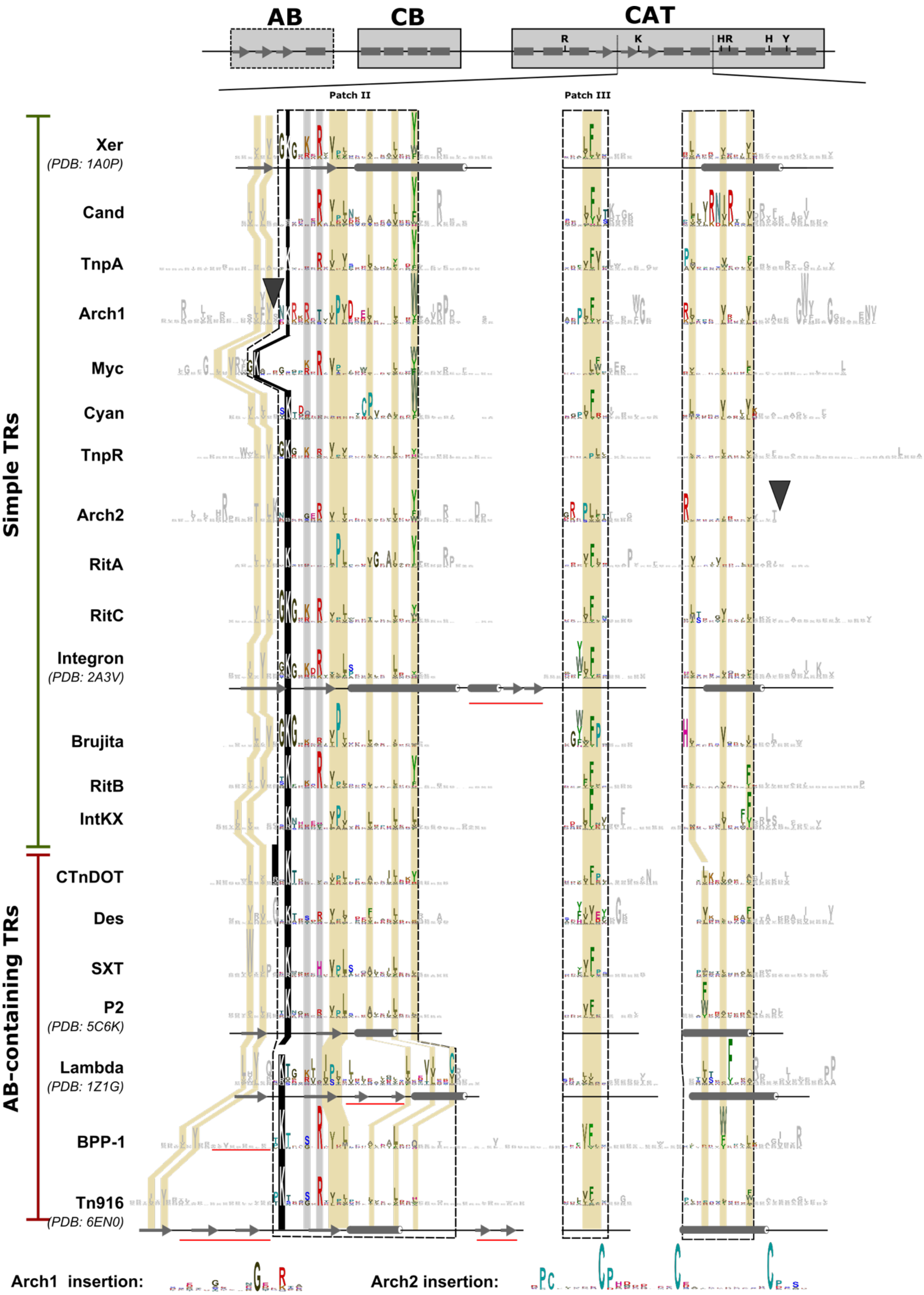

Appendix Figure S3. Sequence conservation of the central part of YR CAT domains. Manually aligned web logos were produced after HMM search against the UniProt reference proteomes database for each of the subgroups. Color code as in Appendix Figure S2. The position of insertions in the Arch1 and Arch2 YRs are indicated with black wedges in the corresponding logos and are shown in full at the bottom of the figure. Insertions in BPP-1, Tn916 and Lambda YRs are underlined in red.

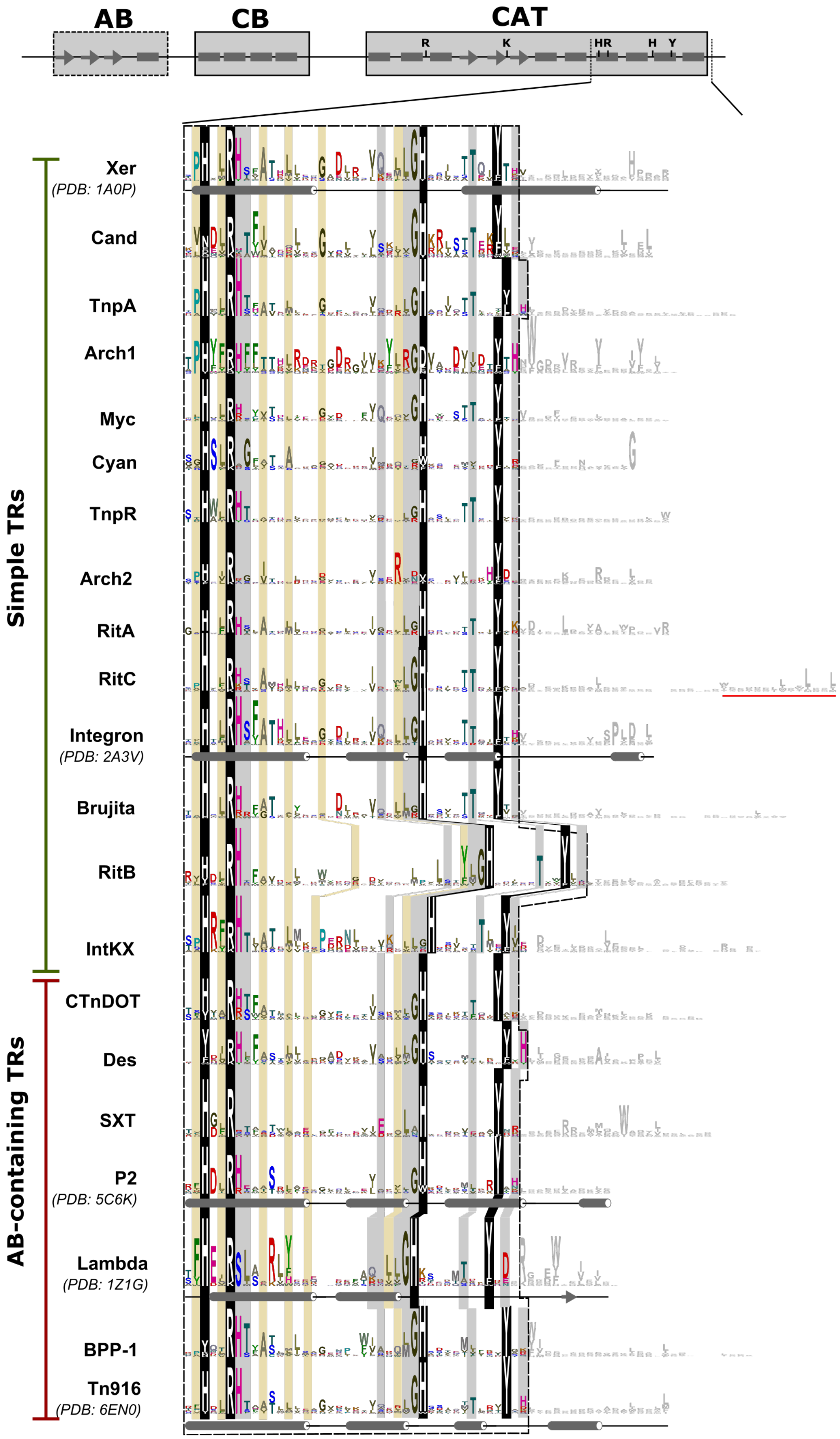

Appendix Figure S4. Sequence conservation of the C-terminal part of YR CAT domain, as in Appendix Figures S2 and S3. Insertions in RitC YRs are underlined in red.

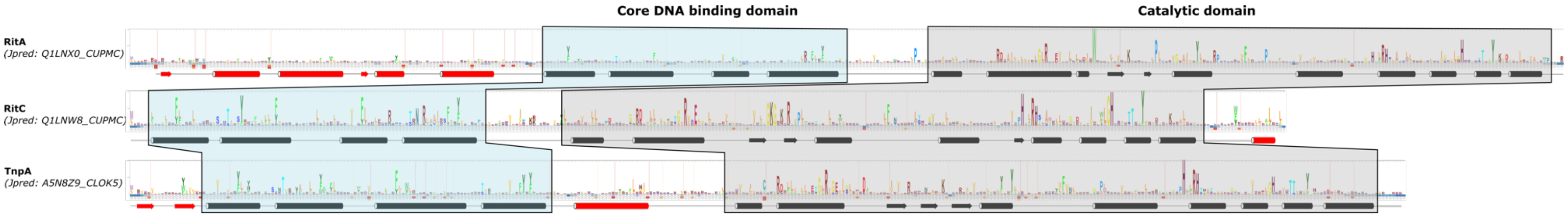

Appendix Figure S5. Full sequence logos of the YRs from RitA, RitC and TnpA subgroups. Different residues are highlighted with different color. Secondary structures were predicted using Jpred and are shown at the bottom of the sequence logos. Secondary structure elements that are specifically conserved within each subgroup and missing in other YRs are highlighted in red.

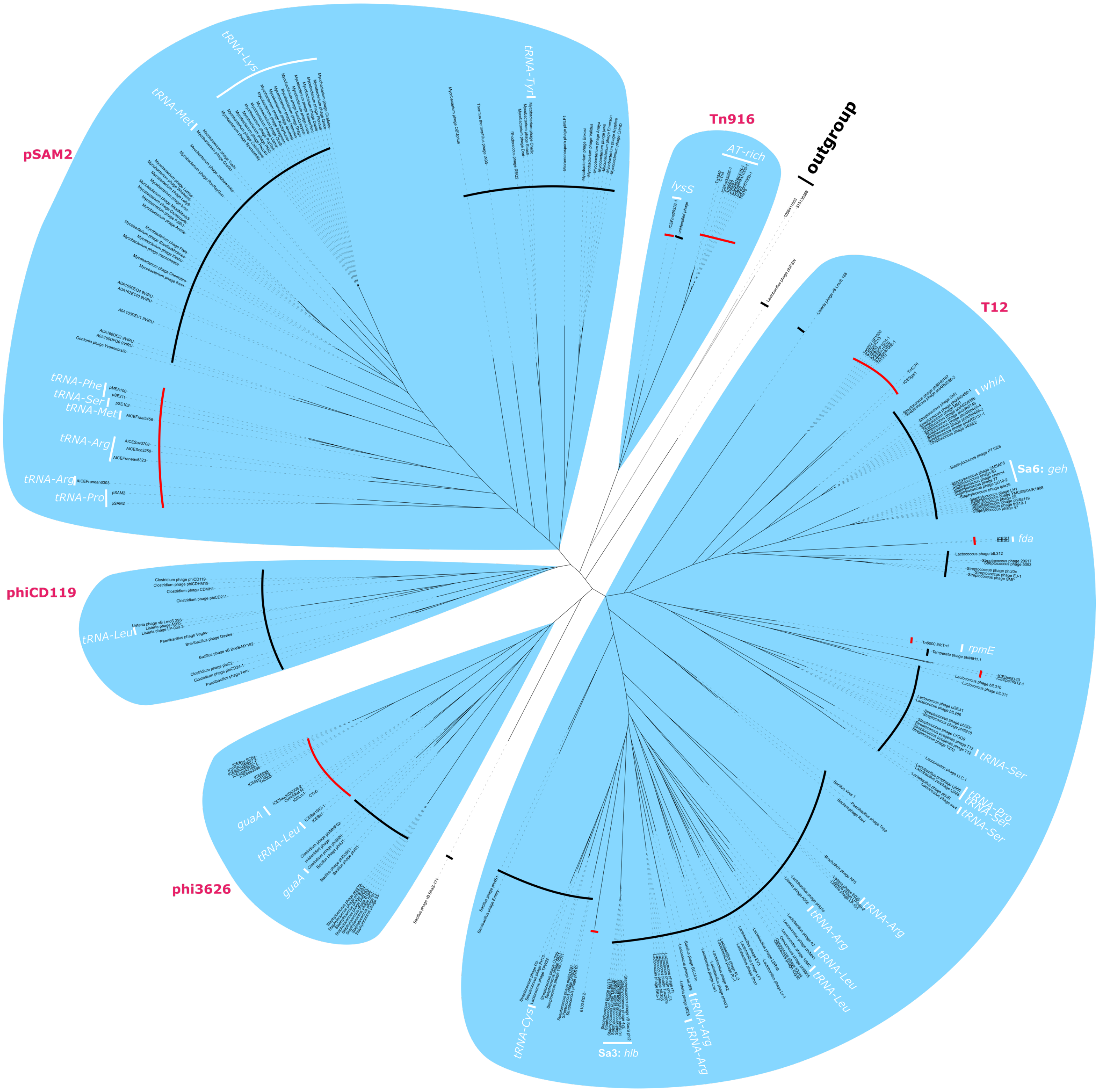

Appendix Figure S6. Maximum-likelihood tree of integrases of ICEs and phages from the Int$_{Tn916}$ subgroup. ICE and phage integrases are denoted by red and black lines inside the leaves (clusters) respectively. Next to each leaf, the name of the cluster is shown in pink, with the characteristic insertion site of its members (if present) indicated with white letters inside the leaves.

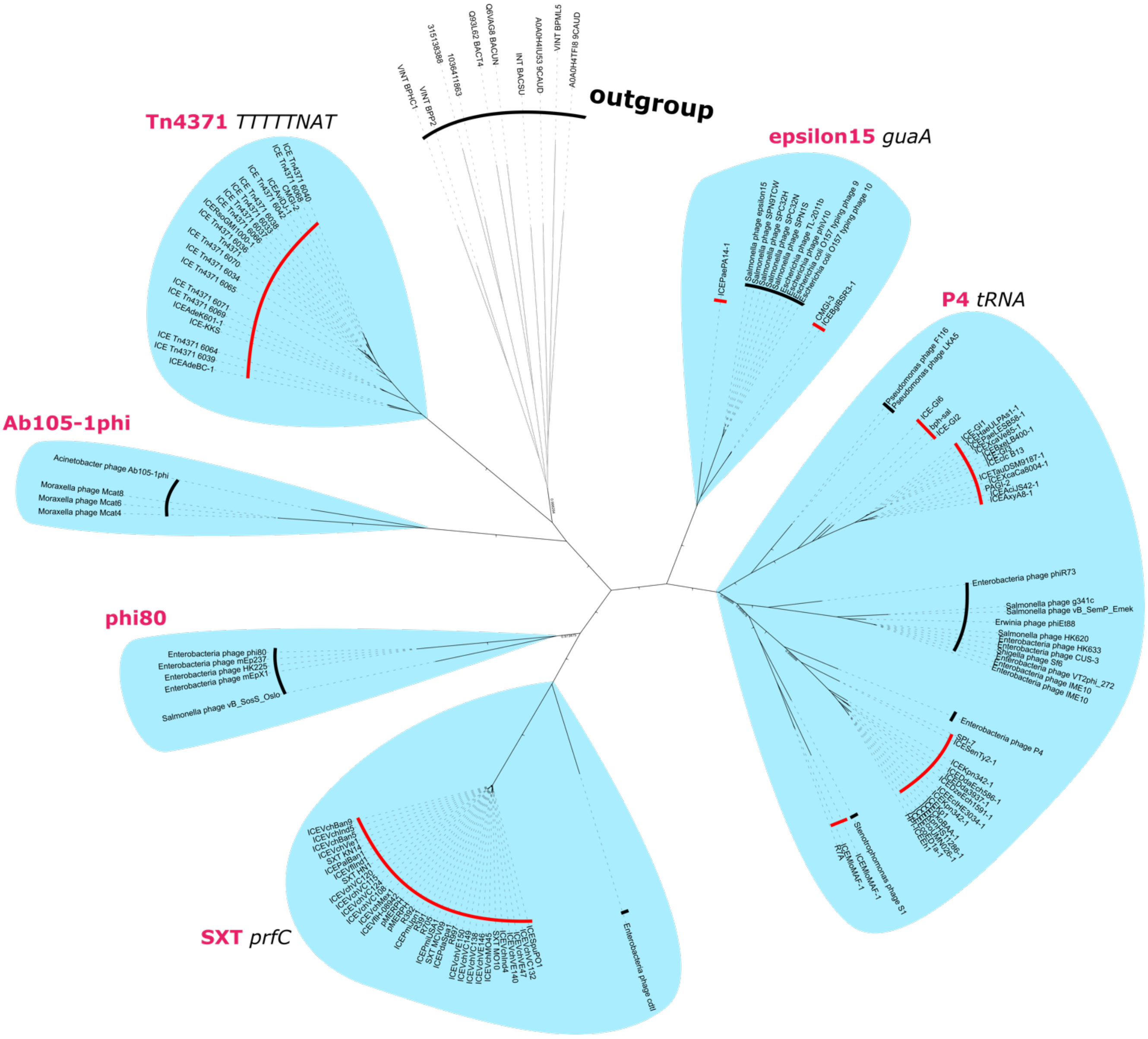

Appendix Figure S7. Maximum-likelihood tree of integrases of ICEs and phages from the Int$_{SXT}$ subgroup. ICE and phage integrases are denoted by red and black lines inside the leaves (clusters) respectively. Next to each leaf, the name of the cluster is shown in pink, with the characteristic insertion site of its members (where identified) indicated in black.

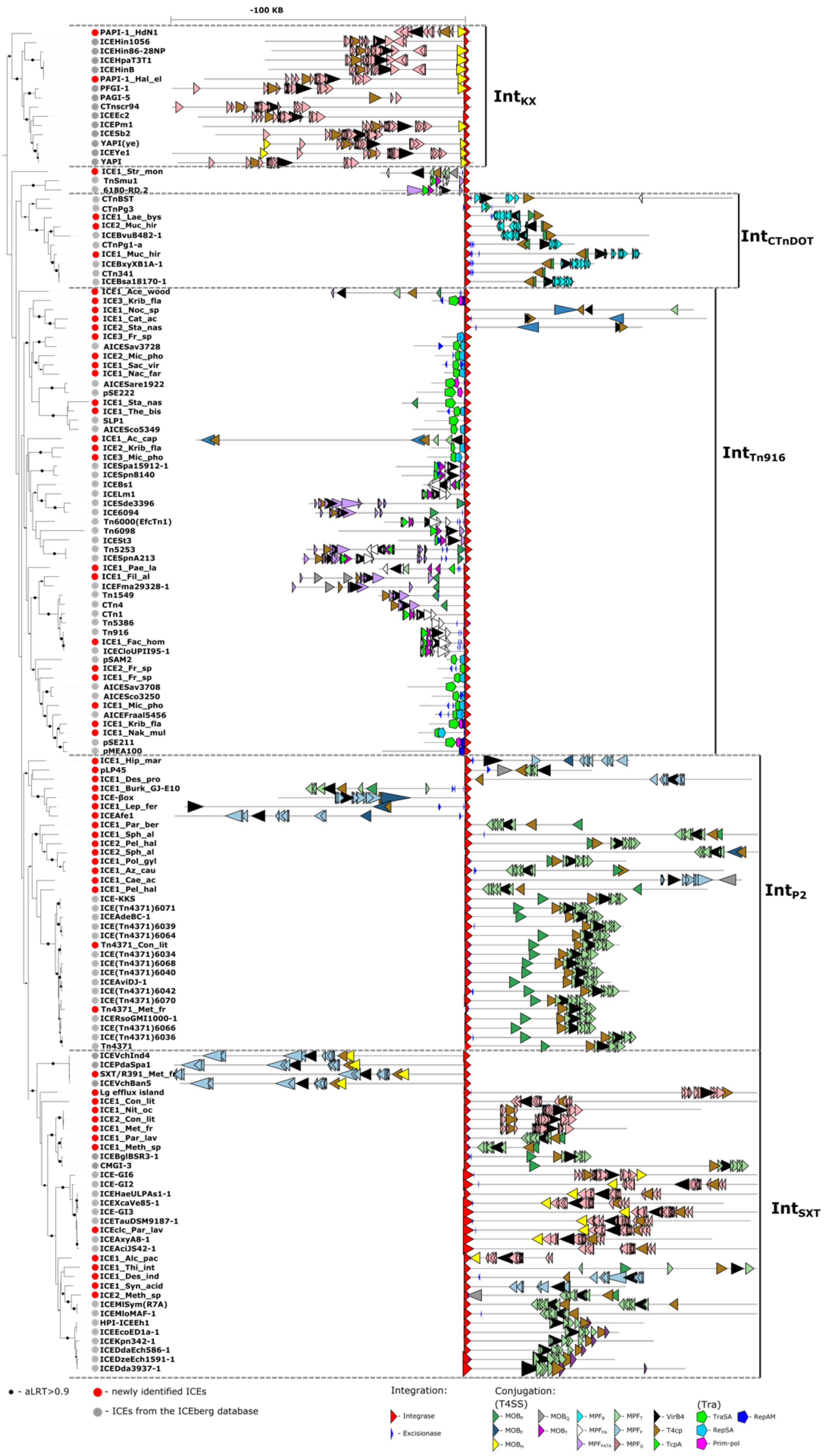

Appendix Figure S8. Structural composition of the ICEs. All ICEs are clustered into five subgroups based on their integrase phylogeny. For that the phylogenetic tree was reconstructed using PhyML as described in the Materials and Methods. New ICEs that were identified in the present study are marked with red circles on the left; ICEs from ICEberg database are marked with grey circles. Schematic representations of ICE architectures are shown, aligned by their integrase genes (red triangle) in the middle. Protein open reading frames of various types of conjugation machineries are depicted with different colours as indicated at the bottom of the figure.

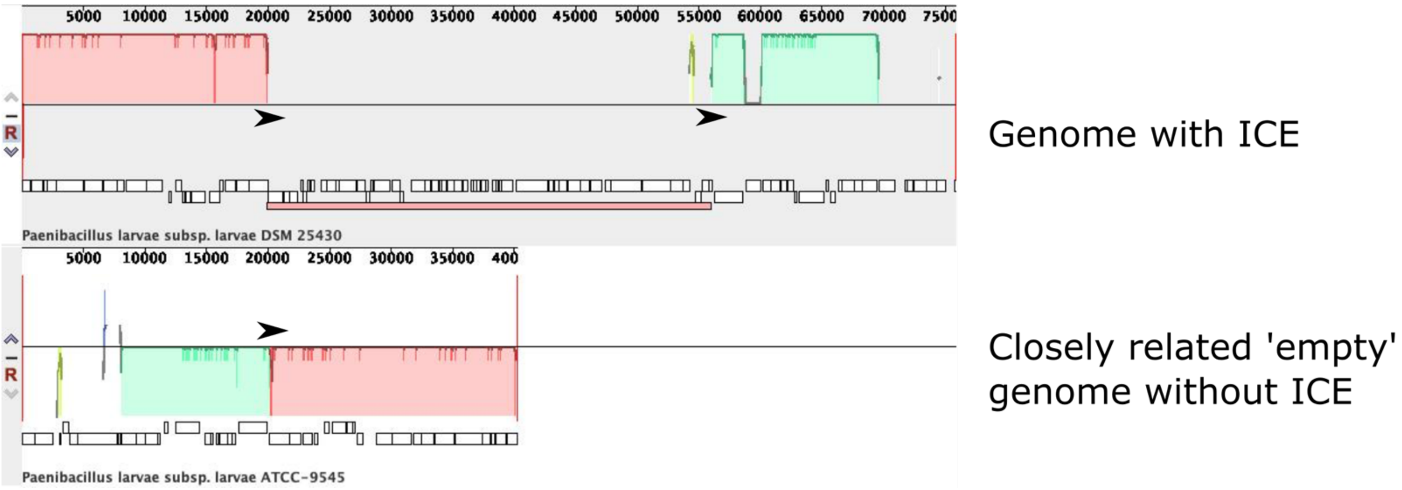

Appendix Figure S9. Verification of the ICE identification. Comparison of two genomes of different *Paenibacillus larvae* strains. The first one (top panel with grey background) contains an insertion of a putative ICE (ICE1_Pae_la, see Dataset EV4). The second one (bottom panel) does not have the ICE insertion and therefore represents a naive site prior the integration of the ICE. The predicted ICE is shown as a pink rectangle in the bottom of the panel. All open readings from the genomic region are shown as white rectangles. The identified terminal repeats are show as black arrows (not in scale). Only one repeat is found in the empty site. The regions of homology are shown as similarity plots with red and green upstream and downstream of the insertion site, respectively. The genome accession numbers as well as positions and sequences of the repeats for all of the identified ICEs are shown in Dataset EV4. The image was produced using Mauve multiple genome alignment tool (http://darlinglab.org/mauve/mauve.html).

Phylogenetic groups	***aBayes***	***SH-aLRT***
Arch1	0.99	0.99
Arch2	1	1
BPP-1	0.99	0.86
Brujita	0.99	0.9
TnpR	1	0.99
Cand	0.99	1
Cyan	0.99	0.95
CTnDOT	1	1
Des	1	1
Integron	0.99	0.86
IntKX	1	0.99
Myc	0.99	0.96
P2	0.98	0.85
RitA	1	0.99
RitB	1	1
RitC	0.99	0.99
SXT	0.99	0.95
Tn916	0.99	0.95
TnpA	0.99	0.93
Xer	0.99	0.92
simple YRs	0.99	0.93

Appendix Table S1. Statistical support for identified phylogenetic clades.

	Pfam	*1.00E-05*	*1.00E-10*	*1.00E-15*	*1.00E-20*	*1.00E-25*	*1.00E-30*	*1.00E-35*	*1.00E-40*
Bacteria	5005	5530	5268	5024	4799	4496	4032	3445	3026
Archaea	137	180	155	136	123	100	70	59	49
Eukaryota	300	1411	756	198	54	24	17	13	9
Viruses	1268	1330	1291	1261	1196	1160	1020	825	742
ICEberg	337	335	327	318	305	298	296	221	191

Appendix Table S2. Benchmarking of e-values for jackhmmer search.
